# Pharmacologic management of HCV treatment in patients with HCV monoinfection vs. HIV/HCV coinfection: Does coinfection really matter?

**DOI:** 10.1371/journal.pone.0225434

**Published:** 2019-11-21

**Authors:** Autumn D. Zuckerman, Andrew Douglas, Kristen Whelchel, Leena Choi, Joshua DeClercq, Cody A. Chastain

**Affiliations:** 1 Specialty Pharmacy Services, Vanderbilt University Medical Center,; Nashville, Tennessee, United States of America; 2 Christy Houston Foundation Drug Information Center, Belmont University College of Pharmacy; Nashville, Tennessee, United States of America; 3 Department of Biostatistics, Vanderbilt University Medical Center,; Nashville, Tennessee, United States of America; 4 Division of Infectious Diseases, Department of Medicine, Vanderbilt University Medical Center,; Nashville, Tennessee, United States of America; Nihon University School of Medicine, JAPAN

## Abstract

**Introduction:**

Sustained virologic response (SVR) rates in patients with hepatitis C virus (HCV) monoinfection and human immunodeficiency virus (HIV)/HCV coinfection treated with direct acting antiviral (DAA) therapy are similar in clinical trials. The objective of this study was to examine differences in patient characteristics, drug-drug interactions, and treatment pathways between these groups in a real-world clinical setting.

**Methods:**

We performed an ambispective review of patients prescribed DAA therapy between September 2015 and April 2018 at a tertiary academic center. The primary endpoint was time from a decision to treat to treatment initiation. Secondary endpoints included patient characteristics; frequency and type of DAA medication interactions; frequency, type, and timing of antiretroviral therapy (ART) changes; and treatment outcomes.

**Results:**

Three hundred and twelve patients were included. Almost half (43%) were HIV/HCV coinfected. Patients with HIV/HCV coinfection were more likely to be African American (p<0.001), have a diagnosed psychiatric disorder (p<0.001) and have a higher pill burden (p = 0.014). Patients with HIV/HCV coinfection were more likely to report an alcohol abuse history (p<0.001), injection drug use history (p<0.024), or active use of illicit substances (p = 0.019). In a multivariable regression model assessing the primary endpoint, time to treatment initiation was increased in patients requiring a change in ART therapy (OR = 9.2, p < 0.001) or a non-ART medication adjustment (OR = 2.4, p = 0.003), and in patients with Medicaid (OR = 6.7, p < 0.001). After controlling for all these factors, HIV/HCV coinfection still significantly impacted time to treatment initiation (OR = 1.7, p = 0.020). The groups had similar rates of drug interaction frequency, treatment completion, observed SVR, and side effects.

**Conclusions:**

Patients with HIV/HCV coinfection are more likely to have a variety of factors that add complexities to HCV treatment. In addition to these challenges, patients with HIV/HCV coinfection experience a longer time to treatment initiation while patients with HCV monoinfection were more frequently lost to care. Care delivery models may incorporate this data to improve patient engagement, access, and outcomes.

## Introduction

Hepatitis C virus (HCV) infection occurs in approximately 2.7 million Americans, causing cirrhosis, end-stage liver disease, and hepatocellular carcinoma in up to 20% of patients with chronic infection.[1] Approximately 5–30% of human immunodeficiency virus (HIV)-infected persons are coinfected with HCV, with higher rates reported in geographic areas where injection drug use is common.[2–5] HIV coinfection accelerates the rate of hepatic fibrosis progression, resulting in more rapid end organ dysfunction in this population. Liver disease, predominantly driven by HCV, remains a leading cause of non-AIDS death in people living with HIV despite the availability of effective HCV treatment.[6–8]

Rates of sustained virologic response (SVR) following HCV direct acting antiviral (DAA) treatment are similar among patients with and without HIV coinfection.[9–11] However, prescribers must navigate treatment complexities of HIV/HCV drug interactions prior to initiating HCV treatment, including potential changes to HIV antiretroviral therapy (ART). ART adjustment often involves coordinated care among multiple providers, including physicians, pharmacists, and social workers.[12] This can impact patients’ ability to initiate HCV treatment in a timely manner, which can be further compounded by arduous medication insurance approval processes.[13]

Though DAA efficacy in HIV/HCV coinfected patients is well established, data are lacking to demonstrate differences in patient characteristics, drug-drug interactions, and treatment pathways among those with HCV monoinfection as compared to HIV/HCV coinfection in real-world settings. Additionally, the frequency at which HIV ART adjustment is required and the subsequent impact on time to HCV treatment initiation has not been comprehensively described. Addressing potential barriers to DAA treatment initiation in patients with HIV/HCV coinfection may facilitate earlier treatment to prevent HCV disease progression.[14]

The purpose of this study was to compare medication management strategies and corresponding outcomes between HCV monoinfected and HIV/HCV coinfected patients treated with DAA therapy in a multidisciplinary infectious diseases clinic.

## Methods

### Setting and study design

We performed an ambispective review of patients seen at the Vanderbilt University Medical Center (VUMC) Infectious Diseases (ID) Clinic and prescribed DAA therapy between September 2015 and April 2018. As previously described in the literature, the VUMC ID Clinic is a multidisciplinary HCV care model involving physicians, a clinical pharmacist, a pharmacy technician, and nurses.[15] Patients are evaluated and counseled by a physician and pharmacist at an initial visit. Most patients have a previous diagnosis of chronic HCV, though this is confirmed by an HCV viral load assessment at time of initial evaluation. During an initial evaluation by a physician and pharmacist, factors that impact treatment decisions are reviewed or collected including HCV genotype, hepatic fibrosis and function, risk factors for HCV acquisition, HCV treatment history (when applicable), and patient readiness to being HCV treatment. Hepatic fibrosis is evaluated using at least one non-invasive staging mechanism such as Fibrosure®, Fibroscan®, or ultrasound with acoustic radiation force impulse (ARFI). The pharmacist provides DAA treatment education, screens for potential drug interactions with DAA therapy, and facilitates medication access and affordability. Referrals for HCV care are from internal and external providers, predominantly working in primary care settings. Once all factors needed for a treatment decision have resulted, the provider and pharmacist determine the best treatment option for a specific patient based on these factors and insurance coverage (this date is termed “decision to treat”). Insurance coverage and financial assistance for DAA treatment are then pursued. After DAA approval by an insurer and the application of financial assistance as needed (termed “DAA approval”), the patient is educated by the pharmacist and therapy is initiated (termed “DAA treatment initiation”).

Following DAA approval by the United States Food and Drug Administration that allowed for interferon-free treatment of HCV, a large cohort of HIV/HCV coinfected patients were internally referred to the VUMC ID clinic from the Vanderbilt Comprehensive Care Clinic, a multidisciplinary clinical program for people living with HIV. Within the VUMC ID clinic, when a change to HIV ART is considered to optimize DAA therapy, the clinical pharmacist or ID clinic physician communicates a recommended ART regimen change to the patient’s HIV provider directly or through a shared electronic health record. The HIV clinic team prescribes and educates patients on new ART. The ID clinic pharmacist subsequently contacts patients to assess adherence and side effects. If the new ART regimen is tolerable and the patient’s HIV viral load remains suppressed (if re-assessed), HCV DAA therapy is initiated. Beyond ART adjustments, HCV monoinfected and HIV/HCV coinfected patients receive the same model of interdisciplinary HCV care and are treated based on the American Association for the Study of Liver Diseases (AASLD)/Infectious Diseases Association (IDSA) Guidance. This study was approved through the Vanderbilt University Investigational Review Board.

Data including patient demographics, disease characteristics such as relevant labs and comorbidities, medication access process such as dates related to the prescription process and insurance type, and treatment outcomes was recorded as part of the electronic health record. Data was then prospectively transcribed by the clinical pharmacist into the REDCap Database housed at VUMC.[16]

### Patient population

Patients prescribed DAA therapy from the VUMC ID clinic whose treatment was approved by third party insurers, medication manufacturer assistance programs, or internal VUMC medication access grants within the study period were included. Definitions of how patients were classified based on disease states or risk factors are shown in Table 1. Patients utilizing manufacturer patient assistance programs or Veterans Administration (VA) benefits for medication acquisition were excluded from time analyses but included in all other analyses. Patients approved for treatment through these programs undergo a significantly different process for obtaining treatment that does not require insurance approval and financial assistance application. Therefore including these patients in the time analysis could skew results.

**Table 1:** Definitions of patient classifications.

**Human Immunodeficiency Virus (HIV) Coinfection- Presence of the following criteria:**
• Prescribed at least three HIV antiretroviral agents • ICD10 of B20
**Cirrhosis–Presence of one of the following criteria:**
• Anatomic ultrasound showing anatomic changes consistent with cirrhosis • Ultrasound with acoustic radiation force impulse predicting F3-F4 or F4 fibrosis • FIB-4 score ≥3.25 FibroSURE^®^ of ≥0.72 • FibroScan^®^ of ≥12.0 kPa (HCV monoinfected) or ≥14.0 kPa (HIV/HCV coinfected) • Liver biopsy with Metavir score F4
**Immunocompromised–Presence of one of the following criteria:**
• CD4 <200 cell/μL • Concurrent treatment with immunomodulators or immunosuppressants • Active lymphoma
**Diagnosed psychiatric disorder**
• ICD10 including F01-F69 and F80-F99
**History of alcohol abuse**
• Patient-reported history of >5 drinks daily on most days of the week
**Active illicit substance or injection drug use**
• Patient-reported use within 3 months of evaluation

The clinic pharmacist screened all patients for medication interactions prior to initiating HCV treatment. The outcome of this review was then classified as one of the following: continue current medications and monitor, dose adjust non-HCV medication(s), substitute non-HCV medication(s), discontinue non-HCV medication(s), change HCV regimen selected, or dose adjust HCV regimen.

### Statistical analysis

The primary endpoint was the time from a decision to treat to treatment initiation, measured in days. Secondary endpoints included baseline demographics and disease characteristics, frequency and type of DAA medication interactions, frequency and type of ART changes, time from treatment approval to ART change, time from ART change to treatment initiation, and treatment outcomes (including side effects, treatment completion and achievement of a sustained virologic response). The primary and secondary endpoints were compared between HCV monoinfected and HIV/HCV coinfected patients.

Categorical variables were described using frequency distributions, while continuous variables were summarized using mean, standard deviation, median, and interquartile range. Proportional odds logistic regression was used to examine the difference in the primary endpoint of time to treatment initiation between the two cohorts. This type of regression is well-suited to outcomes where there is a severe departure from normality, as well as the presence of large outliers, both of which were observed in our data.[17] For a multivariable regression model, in addition to HIV/HCV coinfected status (coinfected vs. monoinfected), covariates were selected *a priori* based on the hypothesis that they may be associated with a change in the time to therapy start: ART change required (yes vs. no), insurance type (Medicaid vs. others), drug interaction management required (excluding ART) (any vs. none), prescription adjustment (excluding ART) (any vs. none), and whether or not the patient was treatment experienced or naïve. For the secondary endpoints, the difference in each endpoint between the two cohorts was assessed using the Pearson chi-square test or Wilcoxon rank-sum test. All analyses were performed with the programming language R version 3.5.1.

## Results

### Baseline characteristics

Three hundred and twelve patients were included in this study. Table 2 describes baseline characteristics. Most were white (66%) and male (68%), with an average age of 52 years. Common HCV characteristics included genotype 1 (80%), treatment naivety (91%), and no-to-mild hepatic fibrosis, F0-F2 (43%). Almost half (43%) were HIV/HCV coinfected. Demographic and social characteristics differed between the groups. Patients with HIV/HCV coinfection were more likely to be African American (p<0.001), have a diagnosed psychiatric disorder (p<0.001), have a higher pill burden (p = 0.014), and were more likely to have an alcohol abuse history (p<0.001), injection drug use history (p<0.024), or active use of illicit substances (p = 0.019). HCV characteristics were similar between the groups. Most patients were treated with ledipasvir/sofosbuvir, including 48% of the HCV monoinfected cohort and 69% of the HIV/HCV coinfected cohort (Table 2).

**Table 2:** Patient demographics.

	Combined N (%) N = 312	HCV Monoinfected N (%) N = 179	HIV/HCV Coinfection N (%) N = 133	P-value
**Age (years) in mean (SD)**	52 (11)	52 (12)	51 (10)	0.064
**Race**				**<0.001**
White	207 (66)	137 (77)	70 (53)	
Black	94 (30)	37 (21)	57 (43)	
Hispanic	4 (1)	1 (1)	3 (2)	
Asian	3 (1)	2 (1)	1 (1)	
Other	4 (1)	2 (1)	2 (2)	
**Male Gender**	211 (68)	111 (62)	100 (75)	**0.014**
**Insurance Type**				**<0.001**
Medicaid	59 (19)	30 (17)	29 (22)	
Medicare	95 (30)	53 (30)	42 (32)	
Private	131 (42)	70 (39)	61 (46)	
Other^a^	27 (9)	26 (15)	1 (1)	
**Genotype**				0.23
1	248 (80)	137 (76)	111 (84)	
2	23 (7)	19 (11)	4 (3)	
3	36 (12)	22 (12)	14 (11)	
4–6 or Multiple	5 (2)	1 (1)	4 (3)	
**Previous Treatment**				0.067
Treatment Naïve^b^	283 (91)	167 (93)	116 (87)	
Treatment Experienced^c^	29 (9)	12 (7)	17 (13)	
**Previous Treatment Regimen**				
Interferon ± ribavirin	26 (81)	12 (80)	14 (82)	
Interferon/ribavirin/telaprevir	1 (3)	1 (7)	0 (0)	
DAA	5 (16)	2(13)	3(18)	
**Fibrosis Score^d^**				0.52
F0-F2	135 (43)	80 (45)	55 (41)	
F2-3, F3	95 (30)	49 (27)	46 (35)	
F3-4, F4	75 (24)	45 (25)	30 (23)	
Unknown	7 (2)	5 (3)	2 (2)	
**Baseline HCV viral load <6,000,000 copies/mL**	227 (73)	134 (75)	93 (70)	0.33
**History of alcohol abuse^e^**	135 (43)	62 (35)	73 (55)	**<0.001**
**History of IDU or illicit substance use^f^**	167 (54)	86 (48)	81 (61)	**0.024**
**Active illicit substance use^e^**	65 (21)	29 (16)	36 (27)	**0.019**
**Psychiatric disorder**	139 (45)	60 (34)	79 (59)	**<0.001**
**Pill burden of non-HCV medications**				**0.014**
0–4	126 (40)	84 (47)	42 (32)	
5–9	85 (27)	47 (26)	38 (29)	
10+	101 (32)	48 (27)	53 (40)	
**HCV DAA Regimen**				
Sofosbuvir/ribavirin	2 (<1)	1 (<1)	1 (<1)	
Ledipasvir/sofosbuvir ± ribavirin	183 (59)	87 (49)	96 (72)	
Velpatasvir/sofosbuvir ± ribavirin	58 (19)	45 (25)	13 (10)	
Dasabuvir/ombitasvir/paritaprevir ± ribavirin	10 (3)	6 (3)	4 (3)	
Daclatasvir/sofosbuvir ± ribavirin	23 (7)	15 (8)	8 (6)	
Grazoprevir/elbasvir ± ribavirin	13 (4)	9 (5)	4 (3)	
Sofosbuvir/velpatasvir/voxilaprevir	3 (1)	0 (0)	3 (2)	
Glecaprevir/pibrentasvir	20 (6)	16 (9)	4 (3)	

^a^Other insurance type included: Veterans Administration CHOICE, Tricare, and self-pay (uninsured)

^b^Treatment naïve is defined as never receiving interferon-based therapies or direct-acting antivirals for the treatment of HCV. DAA: Direct-acting antiviral

^c^Note: There are 29 treatment experienced patients and 32 previous regimens listed as 1 patient had received 3 previous treatments and 1 patient had received 2 previous treatments.

^d^Fibrosis score was determined using non-invasive staging modalities including: Fibrosure®, Fibroscan®, ultrasound with acoustic radiation force impulse (ARFI), APRI, and FIB-4

^e^Alcohol abuse was defined as >5 drinks on most days of the week as reported by the patient

^f^Illicit drug use was considered consumption of illegal substances in the state of Tennessee either through oral, intranasal or inhaled route; DAA: direct-acting antiviral; IDU: Injection Drug Use; HCV: Hepatitis C Virus

### Time to treatment initiation

**Fig 1:**
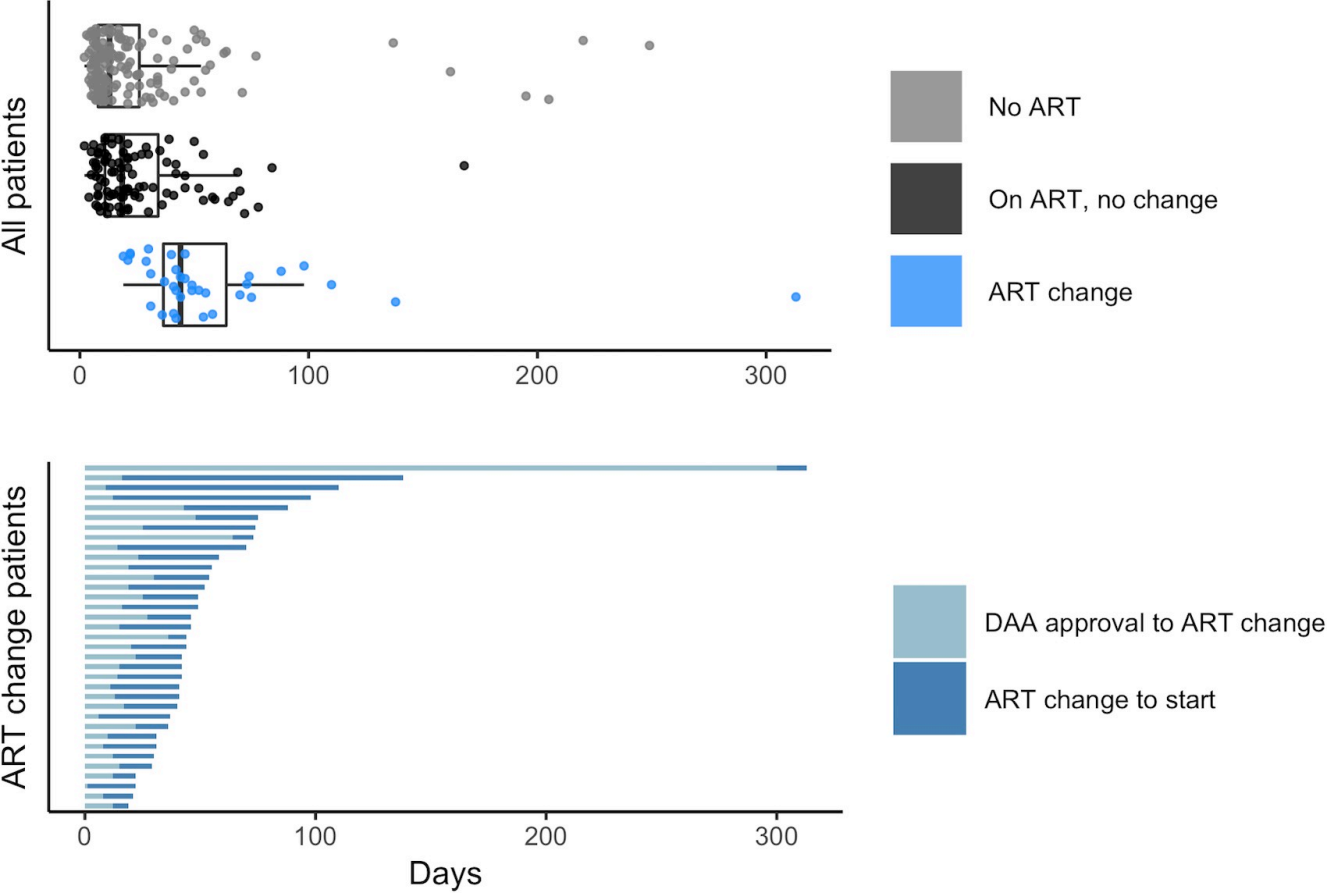
Time to treat by cohort. ART: Antiretroviral Therapy for Human Immunodeficiency Virus; DAA: Direct Acting Antivirals for Hepatitis C Virus; DAA approval refers to insurance approval for treatment Fig 1 displays the variation in time from treatment decision to therapy start. Of the entire cohort, the median time to DAA treatment initiation was lowest in those with HCV monoinfection (13 days), followed by those with HIV/HCV coinfection not requiring an ART change (18 days), and finally patients with HIV/HCV coinfection that did require an ART change (44 days). In HIV/HCV coinfected patients requiring an ART change, the median time from ART change to DAA initiation was longer than the time from DAA approval to ART change, 24 days and 11 days respectively.

All patients included in the study initiated DAA therapy. Thirty-four patients were excluded from our primary endpoint analysis due to use of VA benefits or sole use of a manufacturer patient assistance program for treatment fulfillment as previously noted. Results of the multivariable regression model for the time to treatment initiation are presented in Table 3. Patients requiring a change in ART therapy had a nine-fold increase in the odds of having a longer time to therapy initiation (OR = 9.2, 95% CI = 4.6–18.5, p < 0.001); they had a median of 44 days (IQR 36 to 64) compared to all patients who did not undergo an ART change with a median of 15 days (IQR 9 to 30). Furthermore, the requirement of a medication adjustment outside of ART changes was also associated with a two-fold increase in the odds of delay in therapy initiation (OR = 2.4, 95% CI = 1.4–4.4, p = 0.003). Patients enrolled in Medicaid had much higher odds of having longer time to therapy initiation than patients with any other type of insurance (OR = 6.7, 95% CI = 3.6–12.2, p < 0.001). After controlling for all these other factors, HIV/HCV coinfected patients were still more likely to have a longer time to initiation than HCV monoinfected patients (OR = 1.7, 95% CI = 1.1–2.8, p = 0.020). HCV monoinfected patients initiated DAA treatment in a median of 13 days (IQR 8 to 26), compared to a median of 24 days (IQR 14 to 46) in HIV/HCV coinfection. The median time to treatment initiation in HIV/HCV coinfected patients not undergoing an ART change was 18 days (IQR 11 to 34) (Fig 1).

**Table 3:** Multivariable regression model for the time to treatment initiation.

	Days from BI^a^ to Start Median (IQR)	Odds Ratio (95% CI)	P-Value
HIV/HCV Coinfected		1.7 (1.1–2.8)	**0.020**
Yes	24 (14–46)		
No	13 (8–26)		
ART Change Required		9.2 (4.6–18.5)	**<0.001**
Yes	44 (36–64)		
No	15 (9–30)		
Insurance: Medicaid vs Other		6.7 (3.6–12.2)	**<0.001**
Medicaid	42 (17–65)		
Other	16 (9–30)		
Drug Interaction Management Required (excluding ART interaction)		0.9 (0.5–1.3)	0.483
Any	18 (9–34)		
None	18 (9–41)		
RX adjustment needed (excluding ART Change)		2.4 (1.4–4.4)	**0.003**
Yes	27 (20–41)		
No	14 (9–36)		
Treatment Experienced vs Naïve		0.8 (0.4–1.6)	0.489
Experienced	19 (10–30)		
Naїve	18 (9–38)		

^a^Benefit investigation is defined as a process that enables a provider to determine pharmacy insurance benefit design, coverage requirements and patient out of pocket cost

Median time from decision to treat to DAA insurance approval was similar between the monoinfected and coinfected cohorts, 4 days (IQR 2 to 10) and 4 days (IQR 2 to 12) respectively. Among coinfected patients undergoing an ART change, the median time from approval of HCV medication to ART change was 11 days (IQR 8 to 21), and from ART change to HCV treatment initiation was 24 days (IQR 18 to 33) (Fig 1).

### Medication interactions

Among the study population, 58% screened positive for potential drug interactions at baseline. Both cohorts had a similar prevalence of overall drug interactions, with 55% in the HCV monoinfected cohort and 62% in the HIV/HCV coinfected cohort. The HIV/HCV coinfected cohort had a higher incidence of drug interactions with antiretrovirals and psychiatric medications. (Table 4)

**Table 4:** HCV direct antiviral agent drug interactions and management strategies.

**Rate of Baseline Drug Interactions**			
	**HCV Monoinfected** Number (%) n = 179	**HIV/HCV Coinfected** Number (%) n = 133	
**Drug Interaction Type**			
Acid suppression	50 (28)	27 (20)	
Antiepileptic agents	3 (2)	2 (2)	
Antiretrovirals	0 (0)	46 (35)	
Cardiac agents	29 (16)	12 (9)	
Immunosuppressants	3 (2)	0 (0)	
Antipsychotics	8 (5)	14 (11)	
HMG-CoA reductase inhibitors	27 (15)	20 (15)	
Opioids	6 (3)	3 (2)	
Supplements	2 (1)	1 (1)	
Other^a^	13 (7)	2 (2)	
**Rate of Medication Adjustment Required Prior to Initiating DAA**			
	**HCV Monoinfected** Number (%) n = 98	**HIV/HCV Coinfected** Number (%) n = 82	
**Drug Interaction Type**			
Acid suppression	22 (22)	15 (18)	
Antiepileptic agents	2 (2)	0 (0)	
Antiretrovirals	0 (0)	38 (46)	
Cardiac agents	1 (1)	0 (0)	
Immunosuppressants	1 (1)	0 (0)	
Antipsychotics	3 (3)	4 (5)	
HMG-CoA reductase inhibitors	7 (7)	3 (4)	
Opioids	0 (0)	0 (0)	
Supplements	0 (0)	1 (1)	
Other^a^	4 (4)	0 (0)	
**Drug Interaction Management**			
	**HCV Monoinfected** Number (%) n = 98	**HIV/HCV Coinfected** Number (%) n = 82	**P-value**
**Management Strategies**			
Monitoring	58 (59)	48 (58)	0.93
Non-HCV medication dose adjusted	28 (29)	14 (17)	0.069
Non-HCV medication substituted	7 (7)	38 (46)	**<0.001**
Non-HCV medication discontinued	35 (36)	16 (20)	**0.016**
HCV regimen changed	2 (2)	1 (1)	0.67
HCV regimen dose adjusted	0 (0)	0 (0)	

^a^Other includes: cyclobenzaprine, meloxicam, loperamide, ondansetron, buprenorphine, levothyroxine, fluconazole, rivaroxaban and apixaban

Medication interactions were managed at the following rates among those with a positive screening: monitoring (59%), dose adjustment (23%), substitution (25%), discontinuation (28%) or adjustment to HCV regimen prescribed (2%). Patients may have had more than one medication interaction; therefore these categories are not mutually exclusive. There were no instances where the HCV regimen was dose adjusted. Antiretroviral medications required significantly more changes prior to HCV treatment initiation in the HIV/HCV coinfected cohort (p<0.001). There was a significant difference in the rate of substitution of the non-HCV medication in the HIV/HCV coinfected population (p<0.001), driven by ART changes, and by discontinuation of non-HCV medication in the HCV monoinfected population (p = 0.016). (Table 4) In all HIV/HCV coinfected patients with a non-HCV medication substitution (n = 38), that substitution was an ART change. Most HCV monoinfected patients discontinued acid suppressing agents (74%).

### Changes in HIV ART

**Fig 2:**
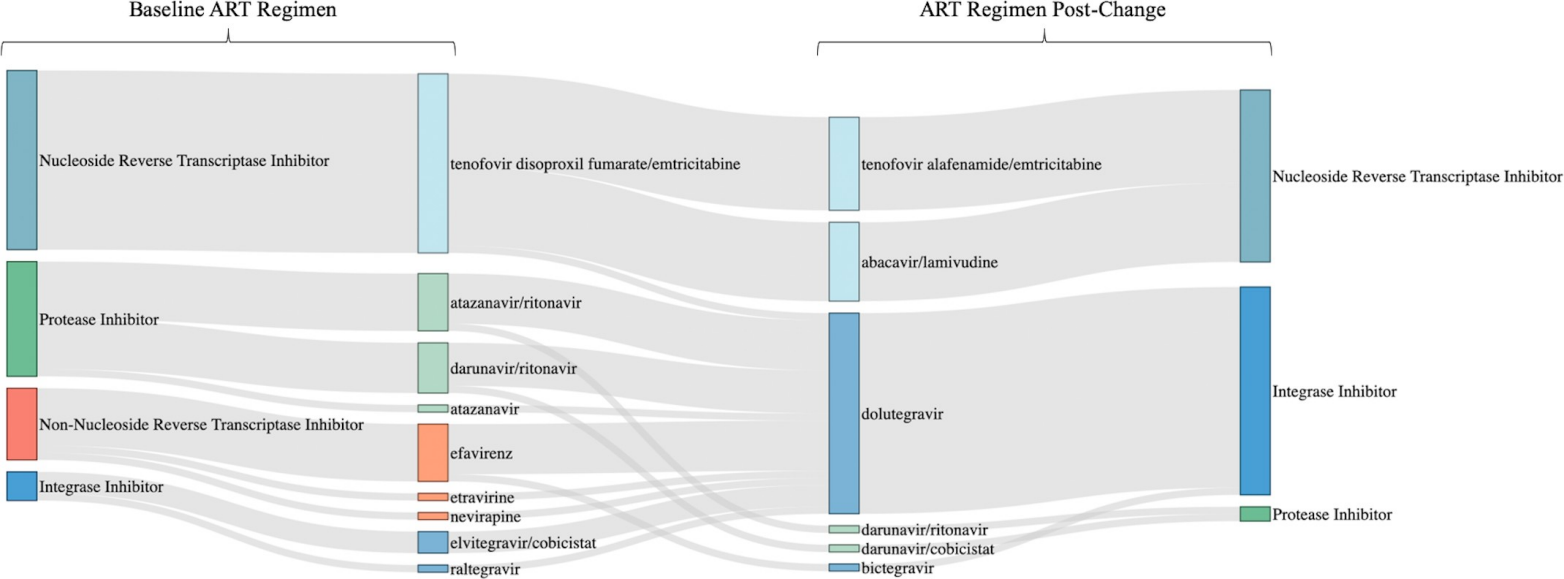
HIV antiretroviral changes. Fig 2 illustrates ART changes made. The left two columns indicate patients’ baseline ART regimen. The right two columns indicate the new ART regimen to which patients were changed. The outer columns are ART regimen classes, while the inner columns are the specific medications utilized within that class. The flow of the diagram shows baseline ART class, baseline ART medication (within those classes), ART medication post-change, and finally ART class post-change. Tenofovir disoproxil fumarate was changed most often to mitigate risk of renal dysfunction with increased tenofovir levels. Most patients were switched from protease inhibitors and non-nucleoside reverse transcriptase inhibitors to integrase inhibitors.

All patients in the HIV/HCV coinfected cohort were on ART prior to initiating DAA therapy for HCV. Of the 133 coinfected patients, a change in ART was made in 38 (29%) patients. Patients who had an ART change were most commonly taking tenofovir disoproxil fumarate (TDF)/emtricitabine (FTC) in combination with a boosted protease inhibitor (61%) or efavirenz (18%) at baseline. As ledipasvir and velpatasvir can both increased TDF concentrations, compounded further by ritonavir or cobicistat coadministration, changes to ART were made to mitigate the risk of renal adverse effects with increased TDF concentrations. Other reasons for an ART change included avoiding dual protease inhibitor use (8%), avoiding potential for hyperbilirubinemia (5%), the opportunity to upgrade an ART regimen to more tolerable agents (3%), and avoidance of a drug interaction with a potential DAA regimen that was ultimately not used (3%). Most patients were switched to an integrase inhibitor regimen (76%). HIV ART changes are detailed in Fig 2. The HCV regimen ledipasvir/sofosbuvir was initiated in 74% of patients post-ART change. All patients whose ART was modified to facilitate HCV treatment continued their new HIV regimen once DAA therapy was completed.

### Treatment outcomes

Each cohort had similar rates of reported side effects (overall 70%). Side effects considered common with HCV DAA therapy (i.e. headache, fatigue and GI disturbances) accounted for 64% of patient-reported side effects overall, with 66% in the HCV monoinfected and 61% in the HIV/HCV coinfected cohort. A total of three patients discontinued HCV treatment due to perceived side effects. One patient discontinued treatment after 3 weeks due to nausea and was lost to follow up. The second discontinued after 10 weeks due to depression and the third discontinued after 7 weeks of treatment due to reported kidney pain; however, these two patients still achieved SVR12. No patients died during treatment.

Documented treatment completion was similar between those with and without HIV/HCV coinfection (98% and 97% respectively) A sustained viral response at least 12 weeks after treatment completion (SVR12) was achieved in 272 (87%) patients. The rate of SVR12 was different between the HCV monoinfected and HIV/HCV coinfected cohorts, 81% and 96%, respectively. However, there were 30 HCV monoinfected patients lost to follow-up prior to SVR12 assessment, with nine having no documentation of treatment completion and 21 who completed treatment but did not provide labs for SVR12 assessment, all of whom were HCV monoinfected. The 30 patients lost to follow up at the end of the study period account for the low observed SVR12 rate in the HCV monoinfected cohort. Excluding patients lost to follow up resulted in SVR12 rate of 98% in the HCV monoinfected cohort.

## Discussion

### Patient characteristics

This study provides insight into the similarities and differences in caring for HCV monoinfected and HIV/HCV coinfected populations. Within our cohorts, baseline demographics including age, gender, HCV genotype, HCV treatment experience, and hepatic fibrosis scores were similar. However, patients with HIV/HCV coinfection were more likely to have social determinants that may negatively impact health, such as alcohol abuse history, IDU history, active illicit substance use, as well as psychiatric disorders. Despite these factors, treatment completion rates in those with HIV/HCV coinfection were similar to those with HCV monoinfection. This aligns with a previous evaluation of the HCV cascade of care which also found that HIV/HCV coinfection did not impact completion of a treatment evaluation following referral to HCV treatment.[15] Therefore, though patients with HIV/HCV coinfection may have more potentially negative social determinants of health, this finding does not seem to impact the ability for HIV/HCV coinfected patients to engage in and benefit from HCV treatment.

### Time to treat

We found a number of significant variables impacting the time to treatment initiation on multivariable analysis including Medicaid insurance, HIV/HCV coinfection, and experiencing an HIV ART change prior to treatment initiation. Among these, having Medicaid insurance caused the most significant delay with a nine-fold increase in the time to treatment initiation. These results add to previous studies demonstrating treatment disparities in patients with Medicaid including a higher rate of absolute treatment denial[18, 19] and longer time to treatment initiation.[15, 20, 21] Delays in treatment initiation in patients with Medicaid have historically been due to significant restrictions to treatment approval based on fibrosis status, drug or alcohol use or history of use, and provider specialty[18]. A previous evaluation of the cascade of care in the VUMC ID clinic found that DAA approval for patients with Medicaid took a median of 30 days (SD 54 ± 73) compared to 4 days in non-Medicaid patients (SD 9±16).[15] A number of advocacy initiatives have highlighted these disparities and have successfully engaged with state Medicaid bodies to reduce requirements for treatment approval, though more work is needed for these requirements to align with AASLD/IDSA Guidance recommendations of treatment for nearly all patients with chronic HCV infection.[22] Providers treating HCV may require dedicated resources to appeal for treatment in patients with Medicaid that are met with treatment restrictions. Letters of medical necessity describing the potential benefits of treatment are sometimes successful in overcoming insurance restrictions.[23] However, these measures contribute to a longer time to treatment initiation.

Controlling for other factors, including HIV ART change, HIV/HCV coinfection was associated with a longer time to treatment initiation (OR 1.7). Though other factors were controlled, additional social determinants may have inadequately measured and disproportionately impacted this population. HCV treatment models should consider these social determinants and optimize services to link patients to care while engaging patients through treatment completion.[24] While a prior study did not identify that HIV coinfected patients waited longer to start HCV treatment, that study’s cohort included only 33 patients with HIV coinfection and had a longer mean time to treatment initiation overall, 30 days in HIV coinfection compared to a median of 24 days in our population.[13]

Requiring an HIV ART modification was associated with a 2.4-fold increase in the odds of having a longer time to treatment initiation. This delay in starting treatment was driven by the time interval following an ART change prior to DAA initiation, in which the pharmacist and the provider monitored the patients for adherence, adverse effects, and other outcomes. The current AASLD/IDSA HCV Guidance does not address when DAAs should be initiated following an ART change. Within our practice, DAA initiation is most often delayed approximately two weeks after an ART change to assess adherence and to monitor for potential adverse effects with a new ART regimen. For patients with significant ART changes that could feasibly impact HIV virologic control, an HIV RNA viral load may be obtained after a longer course of HIV treatment post-ART switch to ensure adequate control prior to DAA initiation. As demonstrated in Fig 2, many patients were changed from a protease inhibitor or non-nucleoside reverse transcriptase inhibitor to an integrase inhibitor. Because the time to DAA initiation can be prolonged in patients requiring an ART change, programs should have a clear mechanism and process of communication to keep patients engaged in care. Additionally, communication among providers involved in HIV and HCV care with clear expectations regarding management and follow-up are essential.

The difference in time to treatment initiation between HCV monoinfected and HIV/HCV coinfected patients is unlikely to change end-organ disease outcomes or mortality over the course of HCV infection. Though the impact of delays in treatment initiation observed in this study may not have had direct effects on clinical outcomes, this disparity is an important finding to help inform HCV treatment practice models. From these results, it is clear that treating HCV infection in patients with HIV/HCV coinfection requires an added level of complexity, especially when an ART change is recommended. Within our clinic, this process was streamlined with the assistance of a clinical pharmacist. Clinics that do not have dedicated resources to navigate medication changes may find a more exaggerated impact on time to HCV treatment initiation in patients with HIV/HCV coinfection.

### Medication interactions

The overall rate of drug interactions was similar between HCV monoinfected and HIV/HCV coinfected patients, even accounting for ART changes. As suggested by other groups, some of the most common drug interactions that were addressed included interactions between DAAs and acid suppression medications as well as cholesterol-lowering therapies.[25, 26] HCV monoinfected patients had a higher rate of non-HCV medication discontinuations as a result of acid reducing therapy being held. Twenty-six of the 35 non-HCV medication discontinuations in the monoinfected cohort were acid reducing agents compared with 16 of the 22 discontinuations in the coinfected cohort. However, HIV/HCV coinfected patients did require more substitutions of non-HCV medication as a result of drug interactions between ART and HCV therapy. Most of the drug interactions only required monitoring, and only 29% of HIV/HCV coinfected patients had an ART modification. The rate and reason for ART change within our cohort was similar to Olea et al. (23% n = 31/135).[12] Of those with an ART change, the most common was adjusting from TDF to tenofovir alafenamide (TAF)-based regimens. As more HIV/HCV coinfected patients are treated with TAF-based therapies prior to consideration of HCV treatment, the need to modify ART specifically for HCV treatment may decrease.

### Clinical outcomes

The rate of reported adverse effects did not differ between HCV monoinfected and HIV/HCV coinfected patients. There was a high incidence of side effects reported (70% overall) compared to clinical trials of common DAA therapies.[27] The high touch multidisciplinary model utilized by the VUMC ID clinic allows for frequent side effect assessment. Patients receiving DAA therapy are contacted three times in the first month of treatment (i.e. 1 week following initiation, week 3 for refill, and week 4 for in-person assessment) and at least once in subsequent treatment months. The high rate of reported adverse effects in this cohort may have been driven by this frequent communication.

High SVR rates in our study population align with other real-world studies of DAA therapy.[11, 12, 28, 29] The difference in SVR rates between our two cohorts was driven by a higher rate of loss to follow up in the HCV monoinfected cohort. Given the higher rate of negative social determinants in HIV/HCV coinfected patients, the lower rate of loss to follow-up was somewhat surprising. A possible hypothesis for these findings is that most HIV/HCV coinfected patients were referred from a comprehensive HIV care clinic and were already engaged in effective HIV care within our healthcare system; therefore, the population referred may have been more likely to remain engaged in our system’s care than HCV monoinfected patients seen from external referral sources. HIV/HCV coinfected patients also had access to additional resources including case management and social work providers that may not be consistently available to HCV monoinfected patients. These findings further establish that HIV/HCV coinfected patients can have similar positive clinical outcomes as HCV monoinfected groups.[29] A growing body of evidence has demonstrated morbidity and mortality benefit in patients with sustained virologic response even in those without advanced liver disease.[30, 31] These benefits may be even more pronounced in patients with HIV/HCV coinfection given the more rapid rate of fibrosis progression in this population. In addition to programmatic interventions on behalf of HIV/HCV coinfected patients, additional resources to encourage retention in care may be helpful in better defining the impact of HCV treatment on all groups, but particularly those with HCV monoinfection that have limited additional healthcare access.

### Implications for HCV programs

These findings can help inform HCV treatment programs, particularly those caring for HIV/HCV coinfected patients. The higher rate of social determinants that may negatively impact health in patients with HIV/HCV coinfection should be taken into consideration and additional resources to address and support these challenges may be advantageous. HCV programs must continue to focus resources on obtaining insurance approval, particularly in those with Medicaid insurance. Medication interactions with DAA therapy are common and should be managed to avoid potential therapeutic ineffectiveness or adverse drug reactions. HIV ART adjustment, occurring in a third of our HIV/HCV coinfected population, should be managed using a clear process that outlines roles, responsibilities, timeline, and communication methods agreed to and executed by a multidisciplinary team to prevent treatment initiation delays. Finally, programs must consider methods to improve retention in care through treatment completion and SVR evaluation in both HCV monoinfected and HIV/HCV coinfected patients to ensure optimal treatment outcomes and post-treatment coordination of care.

#### Limitations

As described, HIV/HCV coinfected patients were referred from a comprehensive multidisciplinary care clinic with resources that may not be reflective of a larger HIV/HCV coinfection population. The sample size is limited. The timeframe of the study and the nature of HCV therapy pipeline in the recent past may impact applicability to other clinical settings.

## Conclusions

Safe and effective DAA therapy has reduced the outcomes gap between HCV monoinfected and HIV/HCV coinfected patients. However, management of HIV/HCV coinfection adds treatment complexities that may impact time to HCV treatment initiation, whether or not an ART change is required. Clear communication between HIV and HCV providers may minimize differences in overall treatment. Pharmacist integration into the care model presents a way to anticipate challenges that may arise concerning drug interactions, facilitate a faster time to initiation by working with medication payers, as well as optimizing outcomes.
